# Second Relapse of Pediatric Patients with Acute Myeloid Leukemia: A Report on Current Treatment Strategies and Outcome of the AML-BFM Study Group

**DOI:** 10.3390/cancers13040789

**Published:** 2021-02-14

**Authors:** Mareike Rasche, Emma Steidel, Martin Zimmermann, Jean-Pierre Bourquin, Heidrun Boztug, Iveta Janotova, E. Anders Kolb, Thomas Lehrnbecher, Nils von Neuhoff, Naghmeh Niktoreh, Nora Mühlegger, Lucie Sramkova, Jan Stary, Christiane Walter, Ursula Creutzig, Michael Dworzak, Dirk Reinhardt

**Affiliations:** 1 Department of Pediatric Hematology-Oncology, Pediatrics III, University Hospital of Essen, 45147 Essen, Germany; Emma.Steidel@uk-essen.de (E.S.); Nils.vonNeuhoff@uk-essen.de (N.v.N.); Naghmeh.Niktoreh@uk-essen.de (N.N.); Christiane.Walter2@uk-essen.de (C.W.); Dirk.Reinhardt@uk-essen.de (D.R.); 2 Department of Pediatric Hematology and Oncology, Hannover Medical School, 30625 Hannover, Germany; zimmermann.martin@mh-hannover.de (M.Z.); ucreutzig@onlinehome.de (U.C.); 3 Division of Pediatric Hematology/Oncology, University Children’s Hospital Zurich, CH-8032 Zurich, Switzerland; Jean-Pierre.Bourquin@kispi.uzh.ch; 4 St Anna Children’s Hospital and Children’s Cancer Research Institute, Department of Pediatrics, Medical University of Vienna, 1090 Vienna, Austria; heidrun.boztug@stanna.at (H.B.); nora.muehlegger@ccri.at (N.M.); michael.dworzak@stanna.at (M.D.); 5 Department of Pediatric Hematology and Oncology, Second Faculty of Medicine, Charles University and University Hospital Motol, 150 06 Prague, Czech Republic; Iveta.Janotova@fnmotol.cz (I.J.); Lucie.Sramkova@fnmotol.cz (L.S.); jan.stary@lfmotol.cuni.cz (J.S.); 6 Nemours/Alfred I. du Pont Hospital for Children, Wilmington, DE 19803, USA; Edward.Kolb@nemours.org; 7 Division for Pediatric Hematology and Oncology, Hospital for Children and Adolescents, University Hospital, Goethe University Frankfurt am Main, 60590 Frankfurt, Germany; Thomas.Lehrnbecher@kgu.de

**Keywords:** acute myeloid leukemia, relapse, childhood acute myeloid leukemia, pediatric, salvage therapy

## Abstract

**Simple Summary:**

Children with acute myeloid leukemia (AML) experience high relapse rates of about 30%; still, survival rates following the first relapse are encouraging. Hence, it is critically important to examine the consequences of a second relapse; however, little is known about this subgroup of patients. This retrospective population-based analysis intends to describe response, survival and prognostic factors relevant for the survival of children with second relapse of AML. Treatment approaches include many different therapeutic regimens, including palliation and intensive treatment with curative intent (63% of the patients). Survival is poor; however, patients who respond to reinduction attempts can be rescued with subsequent hematopoietic stem cell transplantation. We deciphered risk factors, such as short time interval from first to second relapse below one year as being associated with a poor outcome. This analysis will help to improve future international treatment planning and patient care of children with advanced AML.

**Abstract:**

Successful management of relapse is critical to improve outcomes of children with acute myeloid leukemia (AML). We evaluated response, survival and prognostic factors after a second relapse of AML. Among 1222 pediatric patients of the population-based AML-Berlin–Frankfurt–Munster (BFM) study group (2004 until 2017), 73 patients met the quality parameters for inclusion in this study. Central review of source documentation warranted the accuracy of reported data. Treatment approaches included palliation in 17 patients (23%), intensive therapy with curative intent (*n =* 46, 63%) and other regimens (*n =* 10). Twenty-five patients (35%) received hematopoietic stem cell transplantation (HSCT), 21 of whom (88%) had a prior HSCT. Survival was poor, with a five-year probability of overall survival (pOS) of 15 ± 4% and 31 ± 9% following HSCT (*n =* 25). Early second relapse (within one year after first relapse) was associated with dismal outcome (pOS 2 ± 2%, *n =* 44 vs. 33 ± 9%, *n =* 29; *p* < 0.0001). A third complete remission (CR) is required for survival: 31% (*n =* 14) of patients with intensive treatment achieved a third CR with a pOS of 36 ± 13%, while 28 patients (62%) were non-responders (pOS 7 ± 5%). In conclusion, survival is poor but possible, particularly after a late second relapse and an intensive chemotherapy followed by HSCT. This analysis provides a baseline for future treatment planning.

## 1. Introduction

The prognosis of children with acute myeloid leukemia (AML) has improved significantly over the last decades. Current overall survival rates are approaching 70% as a result of intensive frontline treatment, aggressive salvage therapy following relapse and improvements in supportive care [1,2,3,4,5,6,7].

Despite intensive frontline treatments at maximum doses, which include four to five courses of myelosuppressive chemotherapy or an intensive chemotherapy followed by hematopoietic stem cell transplantation (HSCT) for patients categorized as “high-risk,” about 30% of the patients still relapse [8,9]. Facing diminishing returns with further chemotherapy intensification due to toxicity, successful management of relapse is critical to improve outcomes for children with AML while we anticipate the development of new therapies [7,10,11,12,13].

Over the past 20 years, improvements in survival rates are mainly attributed to advances in post-relapse therapy [7]. Since 1987, international reports of survival after first relapse demonstrate a considerable improvement in overall survival for patients in first relapse. The five-year probability of overall survival (pOS) after relapse was 21–24% for patients between 1987 and 1997 [13,14,15], improving to 37–39% in recent studies through 2014 [10,16,17,18,19].

Since 2009, with the completion of the first international relapse AML phase III study for pediatric patients (AML 2001/01 of the International Berlin-Frankfurt-Munster study group), the recommended treatment approach for first relapse includes an anthracycline-based re-induction followed by a second cycle of chemotherapy and HSCT. In Europe, FLAG (fludarabine, cytarabine, granulocyte-colony-stimulating factor) with or without liposomal daunorubicin (DNX) followed by a second course with FLAG has been commonly used [10]. However, there are no specific treatment guidelines for patients experiencing a second relapse.

With improved survival after the first relapse event, it is critically important to examine the consequences of a second relapse in more detail. Published phase I/II studies fail to provide information about general survival and prognostic factors in this subgroup of patients [20,21]. Herein, we report survival results following second relapse from the AML-Berlin–Frankfurt–Munster (BFM) study group from 2004 until 2017, which represents to our knowledge the largest available dataset for this subgroup of pediatric patients. This detailed retrospective analysis intends to describe post-relapse response, survival and any associated prognostic factors relevant for the survival of children with a second relapse of AML.

## 2. Materials and Methods

### 2.1. Patients

Patients with de novo AML enrolled in Germany, Austria, Czech Republic and Switzerland multicenter trials and population-based registries of the AML-BFM study group between 2004 and 2017 (AML-BFM study 2004-ClinicalTrials.gov Identifier: NCT00111345, AML-BFM registry 2012 and AML-BFM study 2012-EudraCT 2013-000018-39) were reviewed. Included were patients between 0–18 years at initial diagnosis. The analysis excluded secondary leukemia, Down syndrome myeloid leukemia, mixed phenotype acute leukemia (MPAL) without AML specific treatment and acute promyelocytic leukemia.

**Figure 1:**
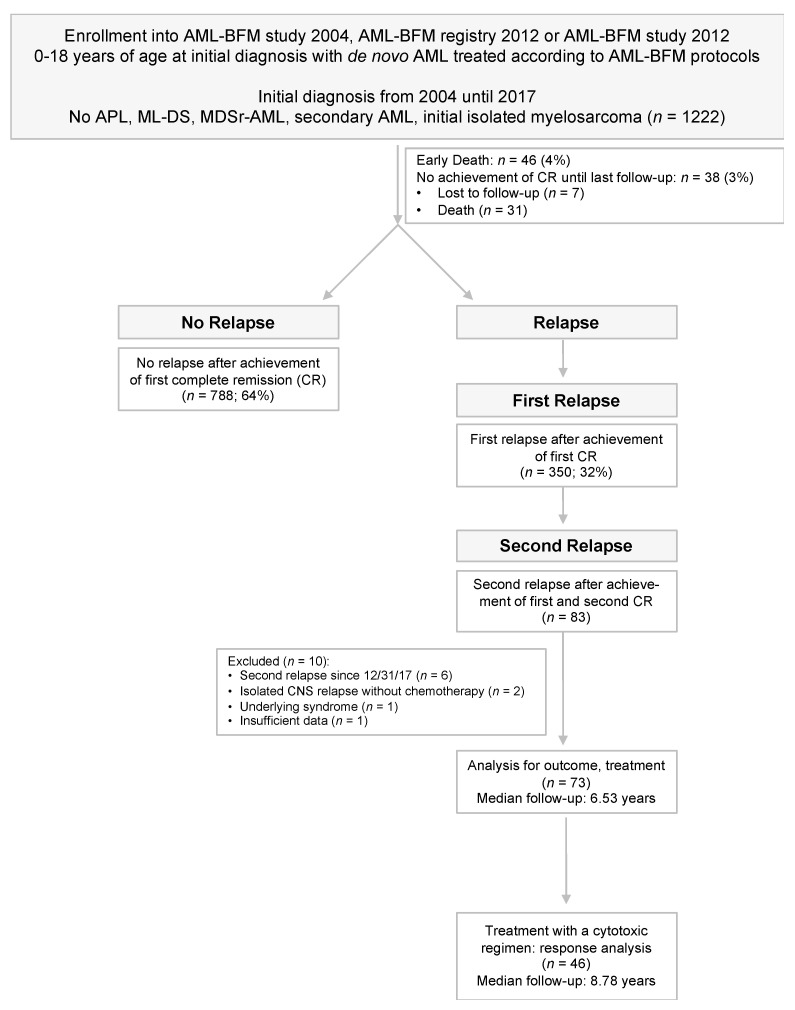
CONSORT diagram. CONSORT flow diagram showing patients of the acute myeloid leukemia- Berlin-Frankfurt-Munster (AML-BFM) studies and registries from 2004 und 2017 that have been included or excluded from the retrospective analysis. Abbreviations: ML-DS, patients with Down syndrome myeloid leukemia; APL, acute promyelocytic leukemia; MDSr-AML, AML with myelodysplasia related changes; CR, complete remission.

Patients less than 22 years of age at second relapse and a documented date of first complete remission (CR1) and CR2 and a date of second relapse between January 2004 and December 2017 were included. We excluded two patients with preceding isolated first CNS relapse who did not receive systemic chemotherapy for the first relapse, one patient with an underlying syndrome and one patient with insufficient data (see Figure 1).

### 2.2. Previous Treatment Approaches

Following initial diagnosis, patients were treated on either the randomized phase III studies AML-BFM 2004 and AML-BFM 2012, or the AML-BFM registry 2012 [4]. All studies were performed after approval by national ethics committees and institutional review boards. Following relapse, 39 patients were enrolled on the Relapse AML 2001/01 trial, which recruited patients from November 2001 to April 2009 [10]. After 2009, treatment guidelines (DNX-FLA, FLA and HSCT) were recommended but were not obligatory and institutional standards governed the treatment of patients. In total, 65 of 73 (89%) patients received (DNX)-FLA following their first relapse.

### 2.3. Definitions and Statistical Analysis

Second relapse was defined as reappearance of leukemic blasts in the peripheral blood, re-infiltration of the bone marrow with ≥5% distinct blasts not to be assigned to any other cause, or distinctive leukemic infiltration elsewhere following CR or partial remission lasting at least four weeks. Reappearance or development of cytologically proven extramedullary disease was considered as relapse. Thus, all patients must have reached two complete remissions before diagnosis of this relapse (CR1 and CR2) (Table S1). Recorded data of the BFM trials and registries including patients with second relapse have been reviewed retrospectively. In addition, medical reports of the treating clinics have been evaluated centrally for detailed information about treatment before and after second relapse, HSCT and cause of deaths. Response assessments following intensive treatment with the intent to induce remission occurred after up to two cycles of treatment. Among 46 patients with intensive treatment, bone marrow response evaluation was available in 45. In *n =* 23 (51%) of these patients, a central review was performed in the national reference laboratories of the BFM study group. Response in the remaining (*n =* 22, 49%) was reviewed at the treating institution. Risk stratification was retrospectively performed for all patients (Table S1).

Statistical analyses were performed with SAS (SAS Institute version 9.4, Cary, NC, USA). All living patients were censored at the time of last follow-up, but not later than 27 March 2020. The median follow-up after diagnosis of second relapse was 6.5 years. Details on definitions are included in Table S1. The Kaplan–Meier method was applied to estimate five-year probabilities of survival and comparisons were performed with the log-rank test. Cumulative incidence functions of early death were constructed according to Kalbfleisch and Prentice. *p* values < 0.05 were considered significant.

## 3. Results

### 3.1. Patient Characteristics

Seventy-three patients fulfilled the inclusion criteria of this study (Table 1). The median age at second relapse was 9.2 years: 8.4 years at first relapse and 7.4 years at initial diagnosis. In total, 60% (*n =* 44) of the patients were male. Forty-four percent (*n =* 32) of patients with second relapse were retrospectively categorized in the “high-risk group” by fulfilling the relevant genetic and response criteria at initial diagnosis. Sixty percent (*n =* 44) of the patients experienced a second relapse within one year after the diagnosis of a first relapse. 

### 3.2. Treatment

Eighty-nine percent of the patients with second relapse (*n =* 65 of 73) had received an anthracycline-containing re-induction (DNX-FLA) followed by FLA or another intensive treatment regimen following the first relapse (see Table 1), and 80% (*n =* 58) and 7% (*n =* 5) had one or two previous HSCTs, respectively. Two patients had a HSCT during first-line treatment only (Table 1). Five patients received a HSCT twice for first-line and relapse therapy. Nine patients did not have any preceding HSCT (Table 1). In contrast to the standardized treatment approaches in the first relapse, patients with a second relapse received a wide range of therapy. We assigned patients to one of three categories: (1) patients with an intensive treatment approach with the intent to induce remission, (2) patients with palliative treatment, or (3) other treatment approaches (see Table 2).

Of 46 patients (63%) receiving intensive systemic therapy, 12 received at least one course of (DNX)-FLA(G) (16%) (Table 2). Three of those patients received (DNX)-FLA(G) in combination with gemtuzumab ozogamicin, and another three in combination with clofarabine. Twenty patients (27%) received no (DNX)-FLA(G), but a gemtuzumab- or clofarabine-based treatment. Fourteen additional patients (19%) received an intensive treatment approach with the intent to induce remission via other individual approaches with or without subsequent HSCT. Seventeen patients (23%) received palliation only (Table 2). Thirteen (76%) of the patients treated only with palliation experienced a second relapse within a year of the first relapse, and 14 patients (88%) had a first relapse within a year of diagnosis. Eleven patients (65%) had both an early first and early second relapse.

The treatment of ten patients was classified as “other”: this category included patients who proceeded directly to HSCT (*n =* 6, 8%), two patients with therapeutic withdrawal of immunosuppression (3%), one patient who received donor-lymphocyte infusions (DLI) with radiation therapy (1%) and one patient treated via an individual approach with blinatumomab and sorafenib. This patient had MPAL treated according to AML-BFM protocols at initial disease and relapse, but received blinatumomab due to CD19 co-expression at second relapse.

In total, 35% (*n =* 25) of all patients proceeded to HSCT; in 88% (*n =* 21) this was a second HSCT (see Table 2).

### 3.3. Survival

**Figure 2:**
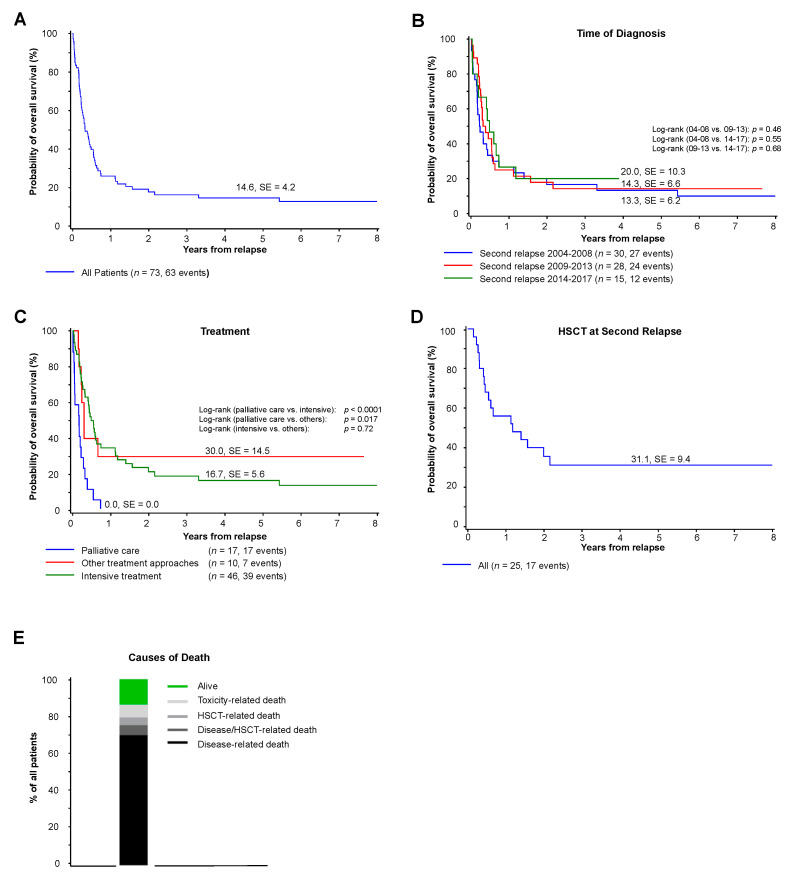
Survival, causes of death and treatment. (**A**) Five-year overall survival in patients with second relapse of pediatric AML diagnosed from 2004 until 2017. (**B**) Five-year overall survival in patients with second relapse of pediatric AML grouped by the time of diagnosis. (**C**) Five-year overall survival in patients with second relapse based on the respective treatment regimen. (**D**) Five-year overall survival of all patients with HSCT after second relapse. (**E**) Cause of death in patients with second relapse classified as disease-related, HSCT-related, toxicity-related or combined causes of death. Abbreviations: HSCT, hematopoietic stem cell transplantation.

Survival after second relapse was poor, with a five-year pOS of 15 ± 4% (see Figure 2A) and a considerable cumulative incidence of early deaths (ED) within the first 56 days after diagnosis of second relapse (cumulative incidence of ED 19 ± 5%). Survival did not improve over time from 2004 to 2017 (see Figure 2B).

As described, patients were treated heterogeneously with approaches including palliation, withdrawal of immunosuppression and several different cytotoxic regimens followed by HSCT. Patients who received an intensive treatment approach achieved a five-year pOS of 17 ± 6%, which rendered a similar outcome as other individualized treatment approaches (n.s.) (see Figure 2C). The median time to death in patients with palliative care was 0.17 years (range 0 to 0.7).

The survival of the 25 patients who received a HSCT was 31 ± 9% (see Figure 2D). Causes of death following a second relapse included disease progression (*n =* 51, 70% of all patients), a combined HSCT-related and disease-related cause (*n =* 3, 5%) and HSCT-related complications (*n =* 4, 4%) or treatment-associated toxicity (*n =* 5, 7%) (see Figure 2E). All ten patients who survived the second relapse previously received DNX-FLA and HSCT after first relapse, but did not receive a HSCT during first-line treatment. In summary, eight of the surviving patients were transplanted at the second relapse. Four of them achieved CR at second relapse before a second HSCT, whereas three were aplastic with no evidence of leukemic blasts before HSCT, achieving a CR only after HSCT. One patient was transplanted with evidence of blasts and received a treatment with sorafenib and DLI afterwards. The remaining two patients without HSCT at second relapse received DLI in combination with other therapies, including one patient who was transplanted subsequently following a third relapse.

### 3.4. Prognostic Factors

**Figure 3:**
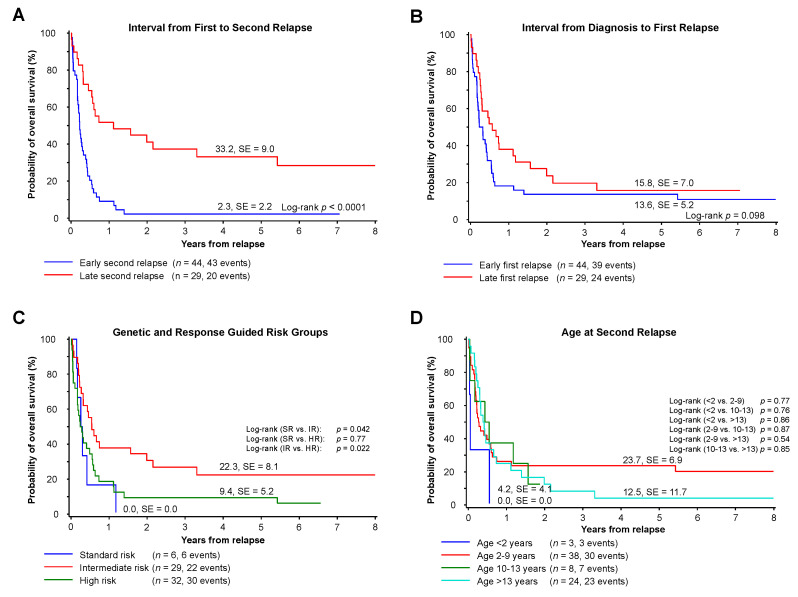
Prognostic factors. (**A**) Five-year overall survival of patients with early or late second relapse defined as relapse within or after one year after first relapse. (**B**) Five-year overall survival of patients with preceding early or late first relapse, defined as first relapse within or after one year after initial diagnosis. (**C**) Five-year overall survival of patients grouped by genetically and response-guided defined risk groups at initial disease. (**D**) Five-year overall survival of patients grouped by age at second relapse.

Probability of five-year survival was 2 ± 2% for patients with an early second relapse (defined as a second relapse within one year after first relapse) vs. 33 ± 9% for those experiencing a second relapse more than one year after the first (*p* < 0.0001; Figure 3A). The time to first relapse did not influence the outcome after second relapse (pOS 14 ± 5% vs. 16 ± 7%, *p* = 0.098; Figure 3B). Survival of patients with high-risk (HR) AML at initial diagnosis was significantly lower compared to intermediate risk (IR) patients (HR 9 ± 5% vs. IR: pOS 22 ± 8%, *p* = 0.022; Figure 3C). Notably, all patients with standard risk (*n =* 6) had an early second relapse and a pOS of 0 ± 0% (Figure 3C). Age at second relapse (<2, 2–9, 10–13 and >13 years) did not show any influence on overall survival (Figure 3D). The pOS was 19 ± 5% for patients with a prior HSCT at first relapse. Patients without any preceding HSCT (*n =* 9), a HSCT during first-line treatment only (*n =* 2) or a HSCT twice for first-line and relapse therapy (*n =* 5) had a pOS of 0 ± 0% (Table 1).

### 3.5. Patients Receiving an Intensive Treatment with the Intent to Induce Remission

**Figure 4:**
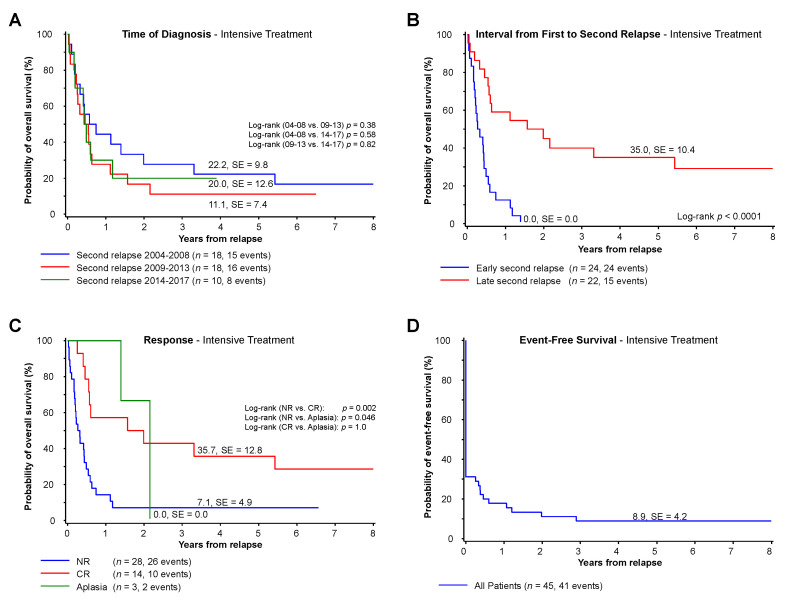
Patients receiving intensive treatment. (**A**) Five-year overall survival in patients with diagnosed second relapse of AML from 2004 until 2017 and treated with intensive treatment grouped by time of diagnosis. (**B**) Five-year overall survival of patients with intensive treatment with early or late second relapse defined as relapse within or after one year after first relapse. (**C**) Five-year overall survival of patients treated with intensive treatment based on the response status after up to two cycles of treatment. (**D**) Five-year event-free survival of patients treated with intensive treatment. Abbreviations: HSCT, hematopoietic stem cell transplantation; NR, nonresponse; CR, complete remission; Aplasia, no evidence of blasts, but missing peripheral regeneration.

All patients receiving intensive systemic treatment with curative intent following a second relapse were analyzed separately (see details in results section “treatment”). As demonstrated in Figure 4A, there was no improvement in survival following therapy for patients with a second relapse of AML treated between 2004 and 2017.

Early second relapse remains a predictor of poor survival (Figure 4B). Risk groups did not reach significance within this smaller subgroup of patients (HR: 13 ± 7% vs. IR: pOS 19 ± 10%, *p* = 0.11).

Forty-five of 46 patients had a best bone marrow response reported for up to two cycles of re-induction therapy. Nearly a third (31%, *n =* 14) achieved a third CR with a pOS of 36 ± 13%, while 62% of the patients showed either a nonresponse (*n =* 28, pOS 7 ± 5%) or no evidence of leukemia with marrow aplasia (7%, *n =* 3, pOS 0 ± 0%; see Figure 4C and Table 2).

Eighteen patients (39%) out of all 46 receiving intensive third-line treatment proceeded to HSCT. Bone marrow response prior to HSCT was available in 17 patients. The pOS of eight patients who achieved a CR prior to HSCT was 38 ± 17%, and 44 ± 22% for the six patients with NEL/aplasia prior to HSCT. None of the three patients with a nonresponse prior to HSCT survived.

In summary, the event-free survival of the 45 evaluable patients was 9 ± 4% at five years (Figure 4D). Despite intensive therapy, 27 patients never achieved a CR, including 25 (54%) patients with continuous blast persistence (pOS 0 ± 0%) and two patients (4%) who continued without visible leukemic blasts and never showed regeneration (pOS 0 ± 0%, see Table 2).

## 4. Discussion

This large retrospective trial confirms that the prognosis of pediatric patients with second relapse of AML remains poor, with a five-year pOS of 15%. Despite encouraging improvements in survival among pediatric patients with AML in the first relapse [10,11,13,14,15,16,17,19,22,23], prognosis of pediatric patients with a second relapse of AML has improved little since 2004. Standardized intensive treatment regimens following an initial relapse yield consistent responses and toxicities, while for patients with a second relapse no general guidelines or trials are available [7]. The development of tolerable and standard regimens for children with a second or greater relapse are needed, as is an international platform for the evaluation of novel therapies.

Patients in second relapse have been heavily pre-treated with several intensive regimens, including at least one HSCT in almost all patients. In this retrospective cohort, therapy following a second relapse included many possible regimens, reflecting the absence of a reliable and effective standard. Intensive therapy including HSCT is required for most patients after a second relapse treated with a curative intent. 

Several prognostic factors are described for patients with first relapse: early treatment response and favorable cytogenetics predict a more beneficial outcome, while an early first relapse, specific molecular high-risk alterations, as well as intense first-line post-remission therapy (HSCT in CR1) translate into poor survival [10,11,14,15,16,19,23,24,25,26,27]. To our knowledge, this report provides the first analysis of potential risk factors at second relapse of pediatric AML. Our data indicate that the interval between the first and second relapse and the response status after treatment and particularly before HSCT have an impact on survival after second relapse. In accordance with previously discussed data on first relapse [14,15,16,19,23,25], we confirmed that a short interval between first and second relapse predicts a poor outcome. This finding underlines the recently published reports that the interval between first HSCT and relapse predicts survival [28]. In our cohort, the interval between diagnosis and first relapse did not impact survival after a second relapse. Remission status following a second relapse and prior to HSCT predicts survival following second relapse. As described for patients with first relapse and patients with relapse after HSCT [7,10,19,28,29,30,31], a subsequent HSCT for patients with a second relapse is required for survival.

Our study did not have a sufficient number of patients to identify cytogenetic or molecular subgroup association with survival following a second relapse. However, the features associated with poor survival after second relapse include a poor treatment response at initial diagnosis combined with genetic high-risk features. Nonetheless, for patients receiving intensive systemic therapy following a second relapse, the combined risk stratification did not reach significance. This is contradictory to what is observed in patients following a first relapse, which may be explained by low patient numbers [10,11,24].

Intense first-line post-remission therapy incorporating HSCT in CR1 has been associated with lower remission and survival rates after first relapse when compared to patients treated with chemotherapy alone [11,13]. Interestingly, a previous HSCT did not impair survival in this analysis of survival following a second relapse.

It is important to consider that patients with second relapse are mainly treated individually and are not included in any clinical trials. Therefore, no systematic data highlighting treatment approaches, response or outcomes exists in the field of pediatric AML. Therefore, it will be crucial for future trials to include a consideration of treatment response. Even though some patients with second relapse are included in several pediatric phase I/II trials, the comparison of response rates is only possible to a limited extent due to the merging of several different refractory disease statuses or the combination with other entities (such as acute lymphoblastic leukemia) in these trials, and the low number of patients included per trial [20,21,29]. Within our subgroup of patients who have been treated with up to two cycles of intensive treatment, the CR rate was 31%.

In a retrospective single-center study in 2000 on adult AML, Stoiser et al. analyzed the prognosis of patients with a second relapse and discussed prognostic factors in these intensively treated patients [32]. Overall, 62 patients were reviewed, of which 33 received further individualized intensive chemotherapy after diagnosis of a second relapse based on the decision of the treating physicians [32]. Eighteen patients (55%) achieved a third complete remission, indicating that intensive chemotherapy after a second relapse might lead to a medium-term survival benefit in adults [32]. Still, this analysis is limited by the single-center design. In addition, differences in terms of pre-treatment (only 16 patients were previously transplanted) and varying inclusion criteria (such as inclusion of patients with PML-RARA) hinder a comparison with our results [32]. 

## 5. Conclusions

In summary, despite the fact that this retrospective data collection to our knowledge represents the largest cohort of children with second relapse of AML, the heterogeneous treatment approaches and non-standardized diagnostic time points limit our analysis. However, even with careful consideration of these limitations, this retrospective cohort will help to implement future trial designs and develop relevant endpoints, as until today no population-based data concerning this subgroup of patients had been published.

## Figures and Tables

**Table 1:** Baseline characteristics.

Characteristics		Second Relapse	Second Relapse Intensive Treatment
Number of patients	*n* (%)	73 (100%)	46 (100%)
Initial characteristics			
Age	(years), median (range)	7.4 (0.2–17.8)	7.7 (0.2–17.8)
Gender	Male	44 (60%)	28 (61%)
	Female	29 (40%)	18 (39%)
FAB	M0	4 (5%)	3 (7%)
	M1/M2	27 (37%)	17 (37%)
	M4/M5	33 (45%)	21 (46%)
	M4eo	1 (1%)	1 (2%)
	M6	2 (3%)	2 (4%)
	M7	5 (7%)	2 (4%)
	Non-classified	1 (1%)	---
Blood counts	WBC (×10^3^/dL) median (range)	13.7 (1.1–331.0)	14.9 (1.1–331.0)
Risk group *	Standard	6 (8%)	3 (7%)
	Intermediate	29 (40%)	18 (39%)
	High	32 (44%)	23 (50%)
	No data	6 (8%)	2 (4%)
Initial response	CR	68 (93%)	43 (94%)
Previous treatment regimen			
Initial treatment protocol	AML-BFM study 2004	62 (85%)	38 (83%)
	AML-BFM registry 2012	9 (12%)	6 (13%)
	AML-BFM study 2012	2 (3%)	2 (4%)
Previous relapse treatment	DNX-FLA(G)+/−FLA(G)	51 (70%)	32 (70%)
	DNX-FLA(G) + other intensive regimen **	14 (19%)	9 (20%)
	Clofarabine-cont. regimen	2 (3%)	2 (4%)
	Others	6 (8%)	3 (7%)
Previous HSCT	No HSCT	9 (12%)	7 (15%)
	HSCT at initial disease only	2 (3%)	1 (2%)
	HSCT at first relapse	56 (77%)	35 (76%)
	HSCT at initial disease and relapse	5 (7%)	3 (7%)
	Unknown	1 (1%)	--
Relapse characteristics			
Age	At first relapse (years), median (range)	8.4 (0.8–18.9)	8.6 (0.8–18.8)
	At second relapse (years), median (range)	9.2 (1.6–20.2)	9.5 (1.8–20.2)
Time to subsequent relapse	Early first relapse	44 (60%)	26 (57%)
	Late first relapse	29 (40%)	20 (44%)
	Early second relapse	44 (60%)	24 (52%)
	Late second relapse	29 (40%)	22 (48%)

* Patients of the AML-BFM study group have been categorized according to the current risk group definition of the last AML-BFM Study (AML-BFM study 2012). It was used prospectively in the AML-BFM registry 2012 and study 2012, while previous patients were analyzed retrospectively for this purpose. Abbreviations: ** All patients have been treated with at least gemtuzumab ozogamicin or clofarabine after DNX-FLA(G). WBC, white blood cells at initial diagnosis; CR, complete remission at initial diagnosis; DNX, liposomal daunorubicin; FLA(G), fludarabine, cytarabine and granulocyte colony-stimulating factor; HSCT, hematopoietic stem cell transplantation; FAB, French–American–British classification at initial diagnosis.

**Table 2:** Treatment and response.

**Second Relapse Treatment and Response of Pediatric AML**		**Patients (%)**
		**73 (100%)**
Chemotherapy *	Intensive treatment with the intent to induce remission	46 (63%)
	• Re-induction including (DNX)-FLA(G) +/−FLA (G)	12 (16%)
	- Additional treatment including GO	3
	- Additional treatment including clofarabine	3
	• Re-Induction including clofarabine or GO, no (DNX)-FLA(G)	20 (27%)
	• Individual approaches	14 (19%)
	Others (including withdrawal immunosuppression, DLI or direct HSCT)	10 (14%)
	Palliative care	17 (23%)
HSCT	No HSCT	47 ^†^ (65%)
	HSCT	25 (35%)
	• First HSCT	2 (8%)
	• Second HSCT	21 (88%)
	• Third HSCT	1 (4%)
	• Unknown	1
	Unknown	1
Response after intensive treatment	Response evaluation available after up to two cycles of therapy	45 (98%)
	• CR	14 (31%)
	• NEL without peripheral regeneration	3 (7%)
	• NR	28 (62%)
	Response evaluation available at the end of treatment including HSCT	46 (100%)
	• CR	19 (41%)
	• NEL without peripheral regeneration	2 (4%)
	• NR	25 (54%)

* Several patients were treated with diverse combinations. For details see results section. ^†^ Including one patient who has been transplanted at the third and one patient at the third and fourth relapse only. Abbreviations: DNX, liposomal daunorubicin; FLA(G), fludarabine, cytarabine and granulocyte colony-stimulating factor; GO, gemtuzumab ozogamicin; DLI, donor lymphocyte infusion; HSCT, hematopoietic stem cell transplantation; NR, nonresponse; CR, complete remission; NEL, no evidence of leukemic blasts, but missing peripheral regeneration. For categories including patients with unknown status, percentages are calculated without “unknown”.

## Data Availability

The data presented in this study are available on request from the corresponding author. A detailed data sharing statement is provided in Supplementary Materials.

## Supplementary Materials

The following are available online at https://www.mdpi.com/2072-6694/13/4/789/s1, Table S1: Definitions. Data sharing statement.
