# Supplementary material for: Second Relapse of Pediatric Patients with Acute Myeloid Leukemia: A Report on Current Treatment Strategies and Outcome of the AML-BFM Study Group
Source: Cancers (Basel). 2021 Feb 14;13(4):789. doi: 10.3390/cancers13040789 (PMC7918758; doi:10.3390/cancers13040789)
Supplement: Supplementary file 1 [file cancers-13-00789-s001.pdf]

Supplemental Table 1. Definitions

**Risk stratification**	
**Standard risk**	Inv(16)(p13.1q22); t(16;16)(p13;q22); t(8;21)(q22;q22); t(1;11) (q21;q23); Normal karyotype and NPM1-mutation; Normal karyotype and CEBPA (double mutation).
**Intermediate risk**	All patients with *de novo* AML, who do not belong to the standard risk group (favorable prognosis) or to the high risk group (unfavorable prognosis). SR and FLT3-ITD.
**High risk**	Abnormalities in chromosome 12p/ t(2;12); monosomy 5/5q-; WT1mut and FLT3-ITD; monosomy 7 (not in combination with favorable/KMT2A-aberrations); t(4;11)(q21;q23); KMT2A/AF4; t(5;11)(q35.3;p15); NUP98/NSD1; t(6;11)(q27;q23)); KMT2A/AF6; t(10;11)(p12;q23); KMT2A/AF10; t(6;9)(p23;q34); t(7;12)(q36;p13); t(9;22)(q34;q11); complex karyotype (≥ three aberrations, at least one structural aberration, without favorable genetics and without KMT2A-rearrangement); inv(3)(q21q26.2)/t(3;3)(q21;q26.2).
**Re-stratification**	Therapy response (evaluated via morphology and immunophenotyping) after the first (~ day 28) and second (~ day 56) induction was used for a subsequent re-stratification. Re-stratification into the intermediate or high risk group is based on nonresponse (≥10% blasts after first or ≥5% after second induction).
**Relapse**	
**Second Relapse**	Reappearance of leukemic blasts in the peripheral blood, re-infiltration of BM with ≥5% distinct blasts (in case of questionable results control after two to three weeks) not to be assigned to any other cause, or distinctive leukemic infiltration elsewhere following CR or partial remission lasting at least four weeks. Reappearance or development of cytologically proven extramedullary disease was considered as relapse. Thus, all patients must have reached two complete remissions before diagnosis of this relapse (CR1 and CR2).
**Response at second relapse**	
**CR**	≤ 5% leukemic blasts in the BM with signs of normal hematopoiesis and with clear signs of regeneration of normal peripheral-blood cell production (platelets > 80 × 10^9/L without transfusions, neutrophils > 1.0 × 10^9/L), and furthermore no leukemic cells in the peripheral blood or anywhere else.
**NEL /Aplasia**	≤ 5% leukemic blasts in the BM in an aplastic BM with no signs of peripheral blood count recovery (platelets < 20 × 10^9/L without transfusions or neutrophils < 0.5 × 10^9/L).
**Nonresponse**	More than 5% of blasts in the BM and/or documented leukemic cells elsewhere after two complete courses of chemotherapy have been considered to be an event at day zero.
**Statistics**	
**pEFS**	Five-year estimates of event-free survival (pEFS) was calculated as the time from diagnosis at second relapse to the next event (third relapse, death of any cause, failure to achieve second remission, or secondary malignancy) or to date of last follow-up. Failure to achieve second remission was defined as an event at day zero, if a patient did not reach CR after two intensive chemo cycles at second relapse.
**pOS**	Five-year estimates of overall survival (pOS) was calculated as time from the date of second relapse to the date of death from any cause or last follow-up.

* Patients have been categorized according to the current risk group definition of the last AML-BFM Study (AML-BFM study 2012). It was used prospectively in AML-BFM registry 2012 and study 2012, while previous patients have been analyzed retrospectively for this purpose.
Abbreviations: BM, bone marrow; CR, complete remission; NEL, no evidence of leukemia; EFS, event-free survival, OS, overall survival.

Data Sharing Statement

The AML-BFM Study Group Data Sharing policy describes the release and use of AML-BFM individual subject data for use in research projects in accordance with EU-Directive of Good Clinical Practice, the guidelines of the German Research Foundation (DFG) and the German Society of Pediatric Oncology and Hematology (GPOH). Only data expressly released from the oversight of the relevant AML-BFM Data and Safety Monitoring Committee (DSMC) are available to be shared. Data sharing will ordinarily be considered only after the primary study manuscript is accepted for publication. For phase III trials, individual-level de-identified datasets that would be sufficient to reproduce results provided in a publication containing the primary study analysis can be requested from the AML-BFM data management. Data are available to researchers who wish to analyze the data in secondary studies to enhance the public health benefit of the original work and agree to the terms and conditions of use. For non-phase III trials, data are available following the primary publication. An individual-level de-identified dataset containing the variables analyzed in the primary results paper can be expected to be available upon request. Requests for access to AML-BFM protocol research data should be sent to the AML-BFM Study Group offices. Data are available to researchers whose proposed analysis is found by the AML-BFM research board to be feasible and of scientific merit and who agree to the terms and conditions of use.

For all requests, no other study documents, including the protocol, will be made available and no end date exists for requests. In addition to above, release of data collected in a clinical trial conducted under a binding collaborative agreement between AML-BFM Study Group and a pharmaceutical/biotechnology company must comply with the data sharing terms of the binding collaborative/contractual agreement and must receive the proper approvals.
